# Evaluating the sustainability, scalability, and replicability of an STH transmission interruption intervention: The DeWorm3 implementation science protocol

**DOI:** 10.1371/journal.pntd.0005988

**Published:** 2018-01-18

**Authors:** Arianna Rubin Means, Sitara S. R. Ajjampur, Robin Bailey, Katya Galactionova, Marie-Claire Gwayi-Chore, Katherine Halliday, Moudachirou Ibikounle, Sanjay Juvekar, Khumbo Kalua, Gagandeep Kang, Pallavi Lele, Adrian J. F. Luty, Rachel Pullan, Rajiv Sarkar, Fabian Schär, Fabrizio Tediosi, Bryan J. Weiner, Elodie Yard, Judd Walson, on behalf of the DeWorm3 Implementation Science Team

**Affiliations:** 1 Department of Global Health, University of Washington, Seattle, United States; 2 Division of Life Sciences, Natural History Museum, London, United Kingdom; 3 Division of Gastrointestinal Sciences, Christian Medical College, Vellore, India; 4 Faculty of Infectious and Tropical Diseases, London School of Hygiene & Tropical Medicine, London, United Kingdom; 5 Department of Epidemiology and Public Health, Swiss Tropical and Public Health Institute, Basel, Switzerland; 6 University of Basel, Basel, Switzerland; 7 Département de Zoologie, Faculté des Sciences et Techniques, Université d'Abomey-Calavi, Cotonou, Benin; 8 Vadu Rural Health Program, KEM Hospital Research Centre, Pune, India; 9 Blantyre Institute for Community Outreach, Lions Sight First Eye Hospital, Blantyre, Malawi; 10 MERIT, IRD, Université Paris, Paris, France; Fundación Mundo Sano, ARGENTINA

## Abstract

Hybrid trials that include both clinical and implementation science outcomes are increasingly relevant for public health researchers that aim to rapidly translate study findings into evidence-based practice. The DeWorm3 Project is a series of hybrid trials testing the feasibility of interrupting the transmission of soil transmitted helminths (STH), while conducting implementation science research that contextualizes clinical research findings and provides guidance on opportunities to optimize delivery of STH interventions. The purpose of DeWorm3 implementation science studies is to ensure rapid and efficient translation of evidence into practice. DeWorm3 will use stakeholder mapping to identify individuals who influence or are influenced by school-based or community-wide mass drug administration (MDA) for STH and to evaluate network dynamics that may affect study outcomes and future policy development. Individual interviews and focus groups will generate the qualitative data needed to identify factors that shape, contextualize, and explain DeWorm3 trial outputs and outcomes. Structural readiness surveys will be used to evaluate the factors that drive health system readiness to implement novel interventions, such as community-wide MDA for STH, in order to target change management activities and identify opportunities for sustaining or scaling the intervention. Process mapping will be used to understand what aspects of the intervention are adaptable across heterogeneous implementation settings and to identify contextually-relevant modifiable bottlenecks that may be addressed to improve the intervention delivery process and to achieve intervention outputs. Lastly, intervention costs and incremental cost-effectiveness will be evaluated to compare the efficiency of community-wide MDA to standard-of-care targeted MDA both over the duration of the trial and over a longer elimination time horizon.

## Author summary

The DeWorm3 Project is a series of randomized clinical trials testing the feasibility of interrupting the transmission of soil-transmitted helminths. We have integrated implementation science research questions into the trials in order to optimize delivery of trial interventions as well as to speed the translation of study evidence into relevant policy and practice. DeWorm3 implementation science research will take place at baseline (formative research), midline (process research), and endline (summative research). DeWorm3 will use stakeholder mapping and network analysis, qualitative data collection via individual interviews and focus groups, structural readiness surveys, process mapping, and economic evaluation methods to assess opportunities to maximize intervention effectiveness, evaluate the efficiency of the intervention relative to the standard-of-care, and identify strategies for sustaining, scaling, and replicating effective components of trial interventions. The implementation science research described in this protocol will be helpful to policy makers and program implementers who aim to use DeWorm3 findings to inform guidelines and routine programmatic activities. The DeWorm3 implementation science protocol is also relevant to researchers interested in incorporating implementation hypotheses into their own clinical research studies.

## Introduction

On average it takes over a decade for clinical research to translate into effective policy and evidence-based practice [1]. However, there is an increased understanding that systematic assessments of the delivery systems, processes, and policies that contextualize clinical research findings are necessary for ensuring that data are relevant and meaningfully adapted to different epidemiological and implementation contexts. In particular, the success of neglected tropical disease (NTD) programs that utilize mass drug administration (MDA) of preventative chemotherapies are highly reliant upon strong distribution channels that facilitate programs achieving high treatment coverage of targeted populations. Given global control and elimination targets outlined in the World Health Organization (WHO) NTD Roadmap and 2012 London Declaration, effective and efficient delivery of MDA programs with high coverage and scale is critical [2].

There are a number of factors that influence MDA coverage in the delivery of routine programs, including drug supply chains, population enumeration, human resource availability and capability, and social sensitization and mobilization. These, in addition to other factors, can be systematically studied through implementation science (IS), which is the scientific practice of identifying, testing and scaling up effective interventions with high quality, fidelity and efficiency. IS is increasingly relevant within NTD programs to ensure that innovations and tools reach populations in need and are appropriately adapted to the local context while maintaining core elements of proven interventional strategies [3]. Applying these methods requires multidisciplinary approaches to studying the process of implementation and the translation of findings into practice. Using IS tools to assess opportunities, address challenges and optimize innovative approaches to NTD control and elimination programs is inherently important and, in light of the Sustainable Development Goals, promotes strong public health delivery systems in NTD-endemic countries [4].

Clinical trials that incorporate implementation or operational research questions are referred to as hybrid studies [5]. The DeWorm3 Project was launched in 2016 as a series of large hybrid trials in Benin, Malawi, and India. The purpose of these trials is to test the feasibility of interrupting the transmission of soil transmitted helminths (STH) using expanded chemotherapeutic approaches while concurrently generating information on the opportunities, challenges, and best-practices for delivering novel STH transmission interruption programs. DeWorm3 aims to preemptively bridge the “know-do” gap by using IS methods to speed the evidence translation process needed to inform STH elimination guidelines, public health practice, and policies and operational plans. By embedding rigorous IS methods within DeWorm3, we aim to (1) describe the implementation environment that contextualizes clinical research findings, and (2) if interventions are found to be efficacious, to generate evidence regarding effective strategies for promoting and scaling-up the interventions to interrupt transmission. This research builds and expands upon prior NTD implementation research from Uganda [6, 7] and Kenya [8, 9] including the ongoing work by the Tumikia project, another randomized trial evaluating the impact of intensified deworming on hookworm transmission [10].

### Aims and objectives

DeWorm3 is a series of cluster randomized trials evaluating the feasibility of interrupting transmission of STH using biannual (twice annually) community-wide MDA targeting eligible community members of all ages. Additionally, DeWorm3 aims to assess the relative influence of community-wide MDA on STH prevalence and transmission intensity as compared to standard-of-care targeted MDA. The rationale, objectives, and design of the DeWorm3 Project clinical trial are described elsewhere in this supplement [11].

**Fig 1:**
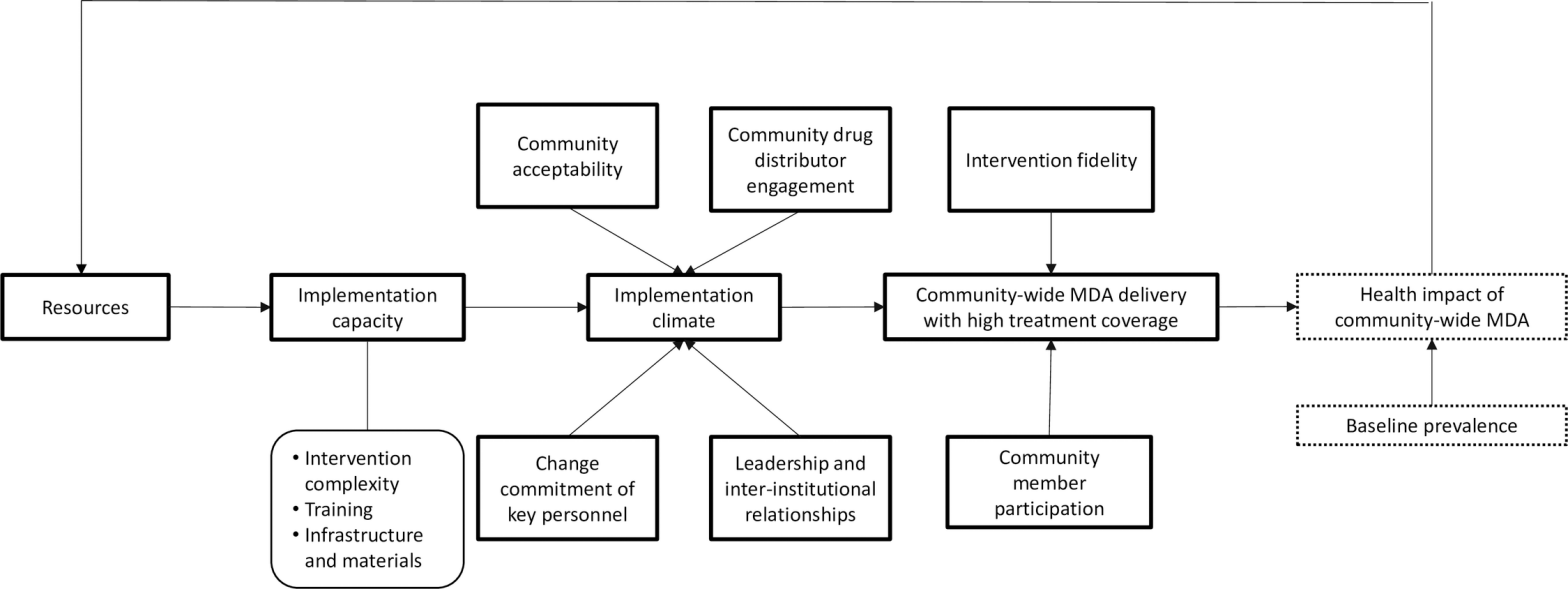
DeWorm3 theoretical model depicting system of influence on successful community-wide MDA delivery.

The effectiveness of community-wide MDA for STH is driven in part by epidemiological factors (baseline disease prevalence and STH species distribution), intervention characteristics (drug efficacy), systems factors (health system strength), and social factors (community member beliefs and preferences) that influence intervention acceptability, penetration, and uptake. These factors are highlighted in the DeWorm3 theoretical model (Fig 1). The overall objective of DeWorm3 IS research is to evaluate these factors in order to develop and test a community-wide STH MDA model that is sustainable and scalable in STH-endemic areas. Specific research aims include:

1. To systematically identify stakeholders influencing standard of care targeted and community-wide MDA and map their potential role and involvement in scale-up of community-wide MDA for STH.
2. To identify implementation-related barriers and facilitators to community-wide MDA for STH from the perspective of various stakeholders:
  - At baseline, to identify barriers and facilitators (i.e. lessons learned) from other community-wide MDA programs, such as lymphatic filariasis (LF) and school-based STH programs, in order to optimize delivery of community-wide MDA for STH.
  - At midline and endline, to assess barriers and facilitators influencing delivery of community-wide MDA for STH and how these barriers and facilitators vary across clusters achieving high and low treatment coverage.
3. To quantify the readiness of the health system to deliver community-wide MDA for STH programs:
  - At baseline, to identify readiness to deliver community-wide MDA for STH programs
  - At endline, to identify readiness to scale-up community-wide MDA for STH programs and assess changes in readiness over time.
4. To map the intervention delivery process and identify any discrepancies between planned and implemented activities in order to optimize the trial intervention.
5. To compare the financial and economic costs and incremental cost-effectiveness of community-wide and targeted MDA for STH in the short- and long-term:
  - To compare the costs and incremental cost-effectiveness of providing six biannual rounds of community-wide MDA versus three annual rounds of targeted standard of care MDA program in each study country.
  - To compare the costs and incremental cost-effectiveness of interrupting STH transmission using a community-wide MDA platform versus a targeted standard of care MDA program in each study country.

## Methods

IS research will be conducted at DeWorm3 baseline (i.e. formative research), midline (i.e. process research), and endline (i.e. summative research). The purpose of conducting formative research is to strengthen DeWorm3 implementation by preemptively identifying implementation barriers and facilitators, seeking support from influential “gatekeepers,” and building consensus. Stakeholder analysis, qualitative data collection, and readiness assessments will contribute to formative research, as described below. Process research will provide important information regarding the contextual factors such as intervention acceptability, uptake, and penetration that influence observed DeWorm3 trial outcomes. Qualitative data collection and process mapping activities will contribute to process research. Lastly, summative research will be used to link implementation factors to key DeWorm3 outcomes (i.e. reductions in prevalence and achievement of transmission interruption) and evaluate how DeWorm3 interventions can be scaled, sustained, or translated into meaningful guideline recommendations. Qualitative data collection, readiness assessments, process mapping, and cost-effectiveness analyses will contribute to summative research. By tracking key implementation factors from baseline and continuously throughout the study, we will be able to identify how the DeWorm3 implementation landscape changes over the duration of the trial.

### Stakeholder analysis

Stakeholder analyses will be conducted in each DeWorm3 trial site at baseline (pre-launch), following trial launch, and at study endline. The purpose of the stakeholder analysis is to identify the individuals who influence or are influenced by effective targeted MDA and community-wide transmission interruption efforts and understand how the network dynamics may influence study outcomes and future policy development. A social network analysis strategy will be used to characterize the relationships between stakeholders in order to (1) describe and map members of the network, (2) characterize the relationship between the network members, and (3) analyze the network using standard network measures such as density, centrality, and homophily [12].

#### Data collection and analysis

A standardized stakeholder mapping exercise will be implemented in all DeWorm3 sites. In the first part of the exercise, working groups composed of the DeWorm3 IS team, personnel from Ministries of Health and Education, and local community-based organizations (CBOs) will identify and describe an exhaustive network of stakeholders who influence or are affected by school-based MDA and, separately, community-wide MDA for STH (adapted from [13]). Working groups will identify stakeholders at each level of the health system, including: the national level; state/regional (or equivalent) level; district (or equivalent) level; local/health center level; community level, including community drug distributors (CDDs), community leaders, and community members; and groups external to the government, including CBOs, non-governmental organizations (NGOs), or international bodies such as WHO.

The stakeholder mapping tool guides the systematic identification of a stakeholder’s influence on the outcomes of the intervention, their attitude towards the intervention, and opportunities to engage the stakeholder throughout the duration of DeWorm3 trial. Additional stakeholders might be added to the network at two follow-up time points, and their attitudes towards the intervention will be updated over time to document changing acceptability of the intervention. During the second part of the exercise, DeWorm3 staff will map the relational power dynamics and dependences between the stakeholders identified by characterizing the directionality of influence (i.e. who influences who) between the stakeholders and the influence category, including: supervision, financial support, technical support, or communication only.

De-identified stakeholder maps in which stakeholder names and affiliations have been removed will be analyzed using standard network measures to understand if: there are stakeholders with extensive influence (i.e. degree centrality), there is high interconnectedness across stakeholders, there are stakeholders who link the network together (i.e. between centrality), there is density around specific stakeholders that might hinder sustainability, there is integration across government ministries and external organizations, or if there is the potential for dysfunctional relationships that might influence intervention effectiveness [12]. Over the duration of the DeWorm3 trials, stakeholder maps will also be used to explore the association between stakeholder attitudes and effective implementation in relevant geographic areas in order to understand if high intervention acceptance of influential stakeholders has a cascade effect at other levels, thereby positively influencing treatment coverage.

### Qualitative research

Qualitative research will be conducted at trial baseline, midline, and endline to identify factors influencing implementation quality, feasibility, and sustainability by site. Qualitative research will utilize the Consolidated Framework for Implementation Research (CFIR) as a guide for data collection and analysis. The CFIR is a validated tool utilized in the United States and increasingly in low- and middle-income countries [14, 15]. The CFIR is built upon a number of preexisting implementation theories to provide a comprehensive meta-theoretical framework of 39 “constructs” that influence implementation [16]. Application of the CFIR helps guide theory development and verify whether and why interventions work through the identification of core and modifiable intervention characteristics [17].

CFIR constructs are organized according to five major domains influencing implementation effectiveness including: (1) the intervention, (2) the inner setting, (3) the outer settings, (4) the individuals involved, and (5) the process of undertaking the intervention. The “intervention” is defined as the core characteristics of the planned intervention. The “inner and outer settings” comprise the contexts where the implementation activities will occur. The “individuals involved” are the agents of change, those who have power and influence to seek, experiment with, or evaluate interventions. Lastly, the implementation process describes an active progression towards attaining the outcome of the intervention described [18]. We have selected a subset of 21 constructs to assess according to the following salience criteria: (1) is the construct a potential barrier or facilitator to community-wide MDA for STH? and (2) Would the construct exhibit heterogeneity across stakeholder groups or clusters? Question guides are designed to address the targeted constructs and are tailored to the stakeholder level (i.e. national, district, etc.). Baseline qualitative research will help refine CFIR construct selection for use during process and summative research as well.

#### Data collection and analysis

Focus group discussions (FGDs) and individual interviews will be conducted with stakeholders from all levels of the health system. FGDs with cluster-level stakeholders will take place in four randomly selected clusters per site, including community members, CDDs, and health center staff or CDD supervisors. FGD participants will be selected using random purposive sampling [19]. Interviews will take place with individuals at higher levels of the health system. These individuals will be selected using purposive quota sampling of mutually exclusive key informant groups (i.e. stakeholder levels) [20].

Trained interviewers who are fluent in local language and customs will conduct one-on-one interviews and small FGDs in private locations with consenting individuals. The interviews will be semi-structured key informant interviews with a mix of respondent and informant style questions. In each site, we use pragmatic criterion to determine the sample size, influenced by the number of levels of the site’s health system. If it is deemed that data saturation has been achieved and redundancy criterion are met within a given stakeholder group after 80% of interviews have been conducted in that group, the sample size may be reduced.

All interviews will be audio recorded and transcribed. Transcriptions will be translated to English. Two primary coders will independently code the site-specific transcripts using CFIR sub-constructs as analysis guides and coordinate their coding to create a comprehensive codebook and thematic analysis. Analyses will be based upon a deductive approach[21]. Special attention will be paid to distinguish factors related to the content and structure of the intervention, opportunities to enhance fidelity of the intervention, penetration within the health system, and opportunities to bring the intervention to scale. Similarities and differences in identified factors will be assessed within and across sites, leveraging the inherent heterogeneity present across clusters.

At midline and endline, qualitative research at the cluster level will take place in four randomly selected clusters per site: two high coverage clusters (80–100% average treatment coverage in year 1) and two low/moderate coverage clusters (<80% average treatment coverage in year 1). CFIR constructs will provide a framework for understanding why the DeWorm3 intervention may exhibit differential effectiveness across settings, correlates of high coverage, and opportunities to optimize the intervention moving forward. Following thematic coding, two independent analysts will assign scores to data at a cluster level to distinguish CFIR constructs that are missing too much data (M), do not distinguish between coverage levels (0), weakly (+1/-1), or strongly (+2/-2) distinguish coverage levels. The ratings reflect the positive or negative influence (valence) and strength of each construct on influencing implementation [18]. These relationships will be evaluated using chi-square tests. Findings will be synthesized to develop recommendations for best practices and highlight barriers and facilitators of implementation in high and low coverage sites.

Quality control will be ensured through a process of (1) random one minute spot checks of each transcribed audio file at a site level, (2) coding review and confirmation of 10% of coded transcripts from each site at the central DeWorm3 level, and (3) coding review and confirmation of 5% of coded transcripts between sites (i.e. circular quality assurance).

### Structural readiness to implement

When a health system is preparing to implement a new intervention or to change standard practice, effective implementation of the change may be influenced by the degree to which stakeholders identify the system as “ready” to implement the change or practice. Within DeWorm3, readiness implies that individuals feel empowered as members of the health system to contribute to the new interventions, they are confident in the flexibility and responsiveness of the system to adapt to the change, they are not threatened by the change, and they believe intervention is appropriate for achieving desired outcomes. With the repurposing of community-wide LF MDA platforms for STH transmission interruption and a transition from targeted to community-wide MDA, there will be a considerable need for systems adaptation and a multi-level and multi-faceted assessment of the government and its partners’ readiness to implement [22].

It is important to understand the factors that drive health system readiness to implement novel interventions such as community-wide MDA for STH both in order to contextualize observed trial outputs and outcomes as well as to provide evidence-based guidance in other settings regarding health system factors that should be in place prior to implementation [23]. Drawing from organizational readiness for change theory, DeWorm3 has developed a structural readiness assessment survey tool [24]. At baseline, the tool will be used to identify needs or conditions that can be targeted for effective change management. At study endline, the readiness tool will be used in a prognostic manner to evaluate health system readiness to sustain or scale-up the intervention [24].

#### Data collection and analysis

The readiness survey instrument aims to capture an individual’s perception of the structural readiness of the health system as well as the downstream phenomena of psychological readiness. Readiness indicators fall into six domains, including: the policy environment, leadership structure, financial resources, material resources, technical capacity, and community delivery infrastructure. Survey questions within each domain are intended to assess the health system’s readiness to launch the new community-wide MDA intervention, deliver community-based public health programs, and function collaboratively. Knowledge assessment questions are used to score a survey participants knowledge of a specific domain, and weight their responses to domain questions accordingly.

The sampling frame for survey participants is defined by the network of individuals identified during baseline stakeholder mapping and updated over time. Participants will be selected using restricted random selection to ensure participation from at least 10% of individuals in each stakeholder group (i.e. national level government staff, CDDs, external partners, etc.), attain sufficient representation from each stakeholder group, and variation across stakeholder groups. Random selection of participants at the local level (health center staff and CDDs) will be restricted to five randomly selected clusters. Surveys will be either self-administered by literate participants or administered in-person by trained study personnel in the local language.

Mean readiness scores and standard deviations will be calculated by site and stakeholder group. We will assess concordance between indicator scores within sites using analysis of variance (ANOVA) models to understand variation in scores amongst and between stakeholder groups.

### Process mapping

Process mapping is a systems analysis approach to identifying the flow of inputs required to achieve optimal outputs, such as high MDA treatment coverage. Process mapping generates a systems-wide view of complex, interdependent components that can contribute to effective MDA programs with high coverage. Process mapping also helps build a shared understanding of how work is carried out and promotes common organizational goals, while simultaneously generating information regarding what aspects of the intervention are adaptable across settings and which are key determinants of intervention uptake.

DeWorm3 will use two process mapping methods. The first is in-depth process mapping, which will take place annually in six randomly selected clusters over the duration of the trial. The second is routine workflow mapping, which will take place in each cluster during each round of MDA. The in-depth process mapping will help identify all activities that must take place in school or community-based MDA programs, *ideal* activity levels for achieving optimized outputs, *actual* activity achievements, and the discrepancy between ideal and actualized activities. Routine workflow tracking will provide information regarding key activity performance in each cluster, and how different activities may or may not be tied to reaching treatment coverage targets. Both of these strategies will identify contextually-relevant modifiable bottlenecks that may be addressed to improve the intervention delivery process and to achieve key intervention outputs.

#### Data collection and analysis

At baseline, implementation science teams in six different clusters per site will engage in guided in-depth process mapping activities. Clusters will be selected based on site-specific indications of capacity. In some sites, this may be historical targeted STH coverage, LF coverage, or immunization coverage. We will use restricted randomization to select two DeWorm3 intervention clusters with high baseline capacity, two intervention clusters with low baseline capacity, one control cluster with high baseline capacity, and one control cluster with low baseline capacity. By including clusters with heterogeneous baseline capacity indications, we augment our ability to observe how the delivery environment responds to or evolves in the context of the DeWorm3 intervention. IS teams in each selected cluster will identify all of the activities and the flow of activities necessary for delivering school-based or community-wide MDA. Ideal metrics and timelines will be identified for each activity. For example, for the activity of sensitization of village leaders, the ideal metric may be that 80% of village headmen with phones are called about an upcoming MDA campaign and the ideal timeline may be one week in advance of drug delivery.

Activity tracking will be ongoing. Once per year, following MDA in both trial arms, selected clusters will record how each mapped activity actually occurred, including the metrics achieved and the relevant time points. This will facilitate analysis of the number of activities that did not meet ideal benchmarks, and if these activities act as bottlenecks to implementing subsequent “downstream” activities. T-tests will be performed to identify if there are changes in the number of activities that do not meet targets within clusters across study years, if certain activity targets are consistently associated with high or low coverage, as well as if there are differences in the number of bottlenecks identified in clusters achieving high or low coverage.

A routine process mapping worksheet will be conducted in all intervention and control clusters during each round of MDA. These worksheets detail activities that must take place in the effective delivery of the intervention. Routine mapping provides opportunities to optimize the intervention in real-time, to systematically measure study inputs and processes, to promote the replicability of the trial, and to identify trial components that may be easily modified or adapted with minimal effects on MDA coverage. Observations from the in-depth process mapping activities will also be used to refine the routine workflow trackers in order to build a useful monitoring tool that may be applicable in other settings.

### Economic research

The financial and economic costs and incremental cost-effectiveness of community-wide and targeted MDA for STH will be ascertained at the end of the DeWorm3 trial (following three years of the intervention and two years of surveillance). Using mathematical models, costs and cost-effectiveness will also be evaluated over a longer time horizon appropriate for an elimination scenario.

#### Data collection and analysis

Inputs required to assess the financial and economic costs of community-wide and targeted MDA for STH will be collected in all DeWorm3 study sites using a standardized cost collection tool. Aligned with economic evaluation guidelines [25–27], the tool follows an ingredients-based micro-costing approach for estimating cost of health interventions; this methodology entails identification and measurement of all inputs required for the intervention, followed by their conversion into monetary value terms to produce a cost estimate. The data will be collected throughout the implementation of the DeWorm3 trial. Data collection rounds will be triggered by site completion of specific trial activities (ex. completing a cross-sectional survey). Aligning data collection with trial activities and routine financial reporting will enhance the quality of the cost data collected by minimizing recall bias and limiting duplication of tasks within the project.

The costs will be assessed from multiple perspectives, namely: individual (i.e. participant), societal, and health systems (i.e. government). Full incremental costs will be derived. Resource use will be valued both in terms of financial and economic costs, including time demanded by community members and Ministry of Health staff to participate in different aspects of the intervention. The intervention costs will be assessed by activity (i.e. community sensitization, population census, MDA, etc.) allowing for greater transparency in reporting and broader generalizability of estimates generated. Within each activity, the contribution of specific, broadly categorized, programmatic inputs will be distinguished (i.e. drugs, wages and per-diems, materials and supplies, equipment and overheads, etc.) This will highlight key cost drivers for MDA interventions. The cost estimates will be summarized annually for each trial arm with total intervention costs calculated by summing annual costs over the duration of the DeWorm3 trial.

The relative efficiency of community-wide and standard of care targeted MDA for STH as implemented in the DeWorm3 trials will be assessed by combining the cost estimates with measures of epidemiological impact of the interventions. A range of outcomes including the percent reduction from baseline to endline prevalence and the total number of prevalent infection case-years averted will be considered to both fully capture the impact of the interventions and allow for comparisons with other studies.

The ultimate goal of DeWorm3 cost-effectiveness research is to provide policy makers and implementers with information regarding the efficiency of community-wide MDA for STH elimination in endemic settings. To this end, the trial will generate key epidemiological and cost data to populate mathematical models of STH epidemiology and control that can also be used to assess the impact of elimination strategies [28, 29]. The models will produce estimates of the incremental impact of community wide MDA for STH for settings outside of the DeWorm3 trial and longer time horizons or 5, 10, 15, 25, and 50 years to appropriately capture STH elimination scenarios. Long-term costs will be discounted and sources of uncertainty will be explored in univariate and probabilistic sensitivity analyses [30, 31].

### Ethics statement

Participants in individual interviews and focus groups will provide written informed consent. Participants who are not literate will sign with a thumbprint in the presence of an impartial witness. Parents of participating children will provide consent on behalf of their child; children will provide assent in accordance with national ethical guidelines. All qualitative data will be confidential and all names or identifying information will be encrypted or removed from transcripts to protect the identity of the participants and their associated institutions.

Written consent is not required for participation in readiness surveys, as approved by ethical review committees. All readiness surveys will be anonymous to protect the identities of participants and their relevant institutions. Only the participant’s affiliated stakeholder group will be recorded.

The IS research component of the DeWorm3 Project has been reviewed and approved by the Institut de Recherche Clinique au Bénin (IRCB) through the National Ethics Committee for Health Research (002-2017/CNERS-MS) from the Ministry of Health in Benin, The London School of Hygiene and Tropical Medicine (12013), The College of Medicine Research Ethics Committee (P.04/17/2161) in Malawi, Christian Medical College in Vellore, and KEM Hospital Research Centre Ethics Committee (1718 and 1719). The study was also approved by The Human Subjects Division at the University of Washington (STUDY00000180).

### Conclusion

The DeWorm3 Project aims to generate additional evidence necessary to optimize the delivery of evidence based interventions tested within the DeWorm3 trials. Regardless of the trial outcomes, these data will contribute to the ability of policy makers and STH programs to deliver high-quality targeted or community-wide MDA at scale. We will utilize recognized dissemination frameworks to ensure that optimal dissemination routes are established early in trial implementation [32]. Results will be disseminated to community members and health workers in trial sites, Ministries of Health in endemic countries, funders, implementing partners, and policymakers at the World Health Organization.

Embedding implementation science methods within the DeWorm3 trial provides an opportunity to study the mechanisms that contribute to acceptable, efficient, and effective community-wide and population targeted MDA. Stakeholder analysis, framework-based qualitative research, structural readiness assessments, process mapping, and cost-effectiveness research will generate the multidisciplinary evidence needed to identify best practices in implementation, core and adaptable components of the intervention across settings, and considerations for sustaining and scaling-up community-wide MDA for STH transmission interruption. Implementing a hybrid trial at such a large scale also provides an opportunity to evaluate opportunities to embed IS into clinical trial work across heterogeneous settings, which may be relevant for other areas of disease focus.

**Editor's Note:** Please note that PLOS Neglected Tropical Diseases does not customarily publish standalone protocols; however, we are pleased to present this protocol as a complement to the research articles in the DeWorm3 collection.

## References

[1] Green LW, Ottoson JM, Garcia C, Hiatt RA. Diffusion theory and knowledge dissemination, utilization, and integration in public health. Annu Rev Public Health. 2009;30:151–74. doi: 10.1146/annurev.publhealth.031308.100049 19705558 10.1146/annurev.publhealth.031308.100049

[2] WHO. Accelerating Work to Overcome the Global Impact of Neglected Tropical Diseases: A Roadmap for Implementation. Geneva World Health Organization, 2012.

[3] Hotez PJ, Pecoul B, Rijal S, Boehme C, Aksoy S, Malecela M, et al Eliminating the neglected tropical diseases: translational science and new technologies. PLoS Negl Trop Dis. 2016;10(3):e0003895 doi: 10.1371/journal.pntd.0003895 26934395 10.1371/journal.pntd.0003895 PMC4774924

[4] Griggs D, Stafford-Smith M, Gaffney O, Rockström J, Öhman MC, Shyamsundar P, et al Policy: Sustainable development goals for people and planet. Nature. 2013;495(7441):305–7. doi: 10.1038/495305a 23518546 10.1038/495305a

[5] Curran GM, Bauer M, Mittman B, Pyne JM, Stetler C. Effectiveness-implementation hybrid designs: combining elements of clinical effectiveness and implementation research to enhance public health impact. Med Care. 2012;50:217–26. doi: 10.1097/MLR.0b013e3182408812 22310560 10.1097/MLR.0b013e3182408812 PMC3731143

[6] Fleming FM, Fenwick A, Tukahebwa EM, Lubanga RG, Namwangye H, Zaramba S, et al Process evaluation of schistosomiasis control in Uganda, 2003 to 2006: perceptions, attitudes and constraints of a national programme. Parasitology. 2009;136(13):1759–69. doi: 10.1017/S0031182009990709 19695107 10.1017/S0031182009990709

[7] Kabatereine N, Fleming F, Thuo W, Tinkitina B, Tukahebwa EM, Fenwick A. Community perceptions, attitude, practices and treatment seeking behaviour for schistosomiasis in L. Victoria islands in Uganda. BMC Res Notes. 2014;7:900 doi: 10.1186/1756-0500-7-900 25495121 10.1186/1756-0500-7-900 PMC4307169

[8] Musuva RM, Matey E, Masaku J, Odhiambo G, Mwende F, Thuita I, et al Lessons from implementing mass drug administration for soil transmitted helminths among pre-school aged children during school based deworming program at the Kenyan coast. BMC Public Health. 2017;17(1):575 doi: 10.1186/s12889-017-4481-7 28615011 10.1186/s12889-017-4481-7 PMC5471907

[9] Macharia JW, Ng'ang'a ZW, Njenga SM. Factors influencing community participation in control and related operational research for urogenital schistosomiasis and soil-transmitted helminths in rural villages of Kwale County, coastal Kenya. Pan Afr Med J. 2016;24:136 doi: 10.11604/pamj.2016.24.136.7878 27642474 10.11604/pamj.2016.24.136.7878 PMC5012741

[10] Brooker SJ, Mwandawiro CS, Halliday KE, Njenga SM, McHaro C, Gichuki PM, et al Interrupting transmission of soil-transmitted helminths: a study protocol for cluster randomised trials evaluating alternative treatment strategies and delivery systems in Kenya. BMJ Open. 2015;5(10):e008950 doi: 10.1136/bmjopen-2015-008950 26482774 10.1136/bmjopen-2015-008950 PMC4611208

[11] Ásbjörnsdóttir KH, Means AR, Schaer F, Yard E, Halliday K, Luty A, et al Assessing the feasibility of elimination of soil-transmitted helminths: the DeWorm3 trial protocol PLoS Negl Trop Dis. 2017. 10.1371/journal.pntd.0006166 PMC5773085 29346377

[12] Blanchet K, James P. How to do (or not to do)… a social network analysis in health systems research. Health Policy Plan. 2011;27(5):438–46. doi: 10.1093/heapol/czr055 21840934 10.1093/heapol/czr055

[13] UNDP. Handbook on Planning, Monitoring, and Evaluating for Development Results New York, NY: United Nations Development Programme, 2009.

[14] Gimbel S, Rustagi AS, Robinson J, Kouyate S, Coutinho J, Nduati R, et al Evaluation of a Systems Analysis and Improvement Approach to Optimize Prevention of Mother-To-Child Transmission of HIV Using the Consolidated Framework for Implementation Research. J Acquir Immune Defic Syndr. 2016;72 Suppl 2:S108–16. 27355497 10.1097/QAI.0000000000001055 PMC5113237

[15] Shelley D, VanDevanter N, Cleland CC, Nguyen L, Nguyen N. Implementing tobacco use treatment guidelines in community health centers in Vietnam. Implement Sci. 2015;10:142 doi: 10.1186/s13012-015-0328-8 26453554 10.1186/s13012-015-0328-8 PMC4600252

[16] Kirk MA, Kelley C, Yankey N, Birken SA, Abadie B, Damschroder L. A systematic review of the use of the Consolidated Framework for Implementation Research. Implement Sci. 2016;11:72 doi: 10.1186/s13012-016-0437-z 27189233 10.1186/s13012-016-0437-z PMC4869309

[17] Damschroder LJ, Aron DC, Keith RE, Kirsh SR, Alexander JA, Lowery JC. Fostering implementation of health services research findings into practice: a consolidated framework for advancing implementation science. Implementation Science. 2009;4:50 doi: 10.1186/1748-5908-4-50 19664226 10.1186/1748-5908-4-50 PMC2736161

[18] Damschroder LJ, Lowery JC. Evaluation of a large-scale weight management program using the consolidated framework for implementation research (CFIR). Implementation Science. 2013;8:51 doi: 10.1186/1748-5908-8-51 23663819 10.1186/1748-5908-8-51 PMC3656778

[19] Bernard H, Ryan G. Analyzing Qualitative Data: Systematic Approaches: SAGE Publications; 2009.

[20] Bernard RH, Ryan GW. Analyzing Qualitative Data: Systematic Approaches: SAGE Publications, Inc.; 2010.

[21] Corbin J, Strauss A. Basics of Qualitative Research 3e. Publications S, editor2008.

[22] Weiner BJ. A theory of organizational readiness for change. Implement Sci. 2009;4:67 doi: 10.1186/1748-5908-4-67 19840381 10.1186/1748-5908-4-67 PMC2770024

[23] Shea CM, Jacobs SR, Esserman DA, Bruce K, Weiner BJ. Organizational readiness for implementing change: a psychometric assessment of a new measure. Implement Sci. 2014;9:7 doi: 10.1186/1748-5908-9-7 24410955 10.1186/1748-5908-9-7 PMC3904699

[24] Helfrich CD, Li YF, Sharp ND, Sales AE. Organizational readiness to change assessment (ORCA): development of an instrument based on the Promoting Action on Research in Health Services (PARIHS) framework. Implement Sci. 4 England2009 p. 38 doi: 10.1186/1748-5908-4-38 19594942 10.1186/1748-5908-4-38 PMC2716295

[25] WHO. Making Choices in Health: WHO Guide to Cost-Effectiveness Analysis. Geneva: WHO, 2003.

[26] MEEP. Methods for Economic Evaluation Project (MEEP): The Gates Reference Case. The Bill & Melinda Gates Foundation, 2014.

[27] Vassall A, Sweeney S, Kahn J, Gomez G, Bollinger L, Marseille E, et al. Reference Case for Global Health Costing: Final Draft for Piloting and Expert Review Following Advisory Group and Stakeholder Meeting 2017.

[28] Tediosi F, Steinmann P, de Savigny D, Tanner M. Developing eradication investment cases for onchocerciasis, lymphatic filariasis, and human African trypanosomiasis: rationale and main challenges. PLoS Negl Trop Dis. 2013;7:e2446 doi: 10.1371/journal.pntd.0002446 24244762 10.1371/journal.pntd.0002446 PMC3820723

[29] Turner HC, Walker M, French MD, Blake IM, Churcher TS, Basanez MG. Neglected tools for neglected diseases: mathematical models in economic evaluations. Trends in parasitology. 2014;30(12):562–70. doi: 10.1016/j.pt.2014.10.001 25455565 10.1016/j.pt.2014.10.001

[30] Drummond M. Methods for the Economic Evaluation of Health Care Programmes. Third ed. New York: Oxford University Press; 2005.

[31] Turner HC, Truscott JE, Hollingsworth TD, Bettis AA, Brooker SJ, Anderson RM. Cost and cost-effectiveness of soil-transmitted helminth treatment programmes: systematic review and research needs. Parasites & vectors. 2015;8:355. 26137945 10.1186/s13071-015-0885-3 PMC4499443

[32] Harris JR. A framework for disseminating evidence-based health promotion practices. Prev Chronic Dis. 2012;9. PMC3277406 22172189

