# Correction: Evaluating the sustainability, scalability, and replicability of an STH transmission interruption intervention: The DeWorm3 implementation science protocol

**DOI:** 10.1371/journal.pntd.0006252

**Published:** 2018-01-31

**Authors:** The PLOS Neglected Tropical Diseases Staff

The affiliation for the seventh author is incorrect. Moudachirou Ibikounle is not affiliated with #5 but with #7 Département de Zoologie, Faculté des Sciences et Techniques, Université d'Abomey-Calavi, Cotonou, Benin.

Additionally, the affiliation for the twelfth author, Adrian J. F. Luty, should appear as: MERIT, IRD, Université Paris 5, Sorbonne Paris Cité, Paris, 75006, France.

The publisher apologizes for the errors.
